# Overnight joint replacement surgery: a pilot Australian study

**DOI:** 10.1111/ans.17977

**Published:** 2022-08-15

**Authors:** Sol Qurashi, Jason Chinnappa, Sam Aktas, Abdul Majid Dabboussi, Md Bayzidur Rahman

**Affiliations:** ^1^ Department Of Orthopaedic Surgery Nepean Hospital Penrith New South Wales Australia; ^2^ Department Of Orthopaedic Surgery Canterbury Hospital Campsie New South Wales Australia; ^3^ The Hip and Knee Clinic Harbourcity Orthopaedics Sydney New South Wales Australia; ^4^ School of Medicine University of Notre Dame Sydney New South Wales Australia; ^5^ Sydney Orthopaedic and Sports Injury Service Sydney New South Wales Australia; ^6^ UNSW Medicine & Health University of New South Wales Sydney New South Wales Australia

**Keywords:** enhanced recovery after surgery, health care rationing, health economics, total hip replacement, total knee replacement

## Abstract

**Background:**

With a stretched healthcare system and elective surgery backlog, measures to improve efficiency and decrease costs associated with surgical procedures need to be prioritized. This study compares the benefits of multi‐disciplinary involvement in an enhanced recovery after surgery (ERAS) protocol‐led overnight model following total hip replacement (THR) and total knee replacement (TKR).

**Methods:**

Patients in each of two private hospitals undergoing THR or TKR were prospectively enrolled. One hospital (Overnight) was fully committed to the ERAS protocol implementation on all levels and formed the treatment group while in the other hospital (control), patients only had the anaesthetic and operative procedure as part of the ERAS protocol but did not follow the perioperative measures of the protocol. Outcomes on hospital length of stay (LOS), inpatient rehabilitation, functional outcomes, satisfaction, adverse events and readmission rates were investigated.

**Results:**

Median LOS in the Overnight group was significantly smaller than in the control group (1 vs. 3 days, *P* < 0.0001). The Overnight group had lower rates of inpatient rehabilitation utilization (4% vs. 41.2%, *P* < 0.0001), similar improvements in functional hip and knee scores and no increased rate of adverse events or readmission. All patients in both groups were satisfied with their treatment.

**Conclusion:**

Overnight THR and TKR can safely be performed in the majority of patients, with a multi‐disciplinary approach protocol and involvement of all perioperative stakeholders.

## Graphical abstract

There are significant public and private elective surgery backlogs as a result of the pandemic measures needed for COVID‐19. Overnight short stay joint replacement is safe and feasible with similar patient functional outcomes as well as satisfaction when an ERAS protocol is implemented with the involvement of all stakeholders. Overnight short‐stay joint replacement surgery will save costs and hospital resources, therefore allowing more patients to undergo surgery within a defined budget.

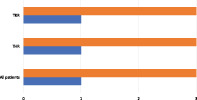

## Introduction

Prior to the COVID‐19 pandemic, backlogs and increasing demand on the already stretched healthcare system was pushing it to the brink of economic unsustainability.^1^ Similar experiences in the USA led to policy change in funding models and concepts such as bundled care.^2^ While the global focus had been slowly shifting to enhanced recovery after surgery (ERAS) protocols and shorter inpatient hospital stays following total joint replacement surgery for hip and knee arthritis for some time, the economic push drove these changes in the US healthcare model a lot faster. This was due to the proven cost saving and patient perceived benefit associated with ERAS models.^3^, ^4^, ^5^

Considering the current situation of cost and waitlists for joint replacement surgery in the Australian setting, especially in the post COVID landscape, it is imperative to harness the benefit of such models for both the patient as well as the system. By getting suitable patients home on the same day or following an overnight stay in hospital, the healthcare system would benefit with increased case capacity (from decreased bed blocks) and significantly decreased costs per case. Patients would benefit from being able to recover in a familiar setting with all the support from allied health services that they would have had as an inpatient as well as support from family and friends, return to remote work sooner and the aforementioned cost savings. Further, evidence in the literature shows no benefit and increased costs from inpatient rehabilitation following all total hip (THR) and total knee (TKR) joint replacement surgeries.^6^, ^7^, ^8^, ^9^ We have previously demonstrated the safety and effectiveness of our ERAS protocol for THR and TKR in public and private Australian hospital settings.^10^

This study aims to compare the benefits of multi‐disciplinary involvement in an ERAS protocol following THR and TKR. We report on the safety of this approach and its effect on overnight joint replacement surgery in an Australian setting through outcomes on hospital length of stay (LOS), inpatient rehabilitation utilization rates, patient functional outcomes, patient satisfaction, adverse events and hospital readmission rates.

## Methods

### Patients

Consecutive patients in each of two private hospitals undergoing THR or TKR under the lead investigator were prospectively enrolled for inclusion in this study. Exclusion criteria were patients with a background history of substance abuse, malignant hyperthermia or allergy to anaesthetic agents, patients with impaired cognitive function and revision surgery procedures.

### Study design

The two private hospitals where patients underwent surgery had different goals and practices regarding perioperative patient care which formed the basis of the different groups. One of the two hospitals was fully committed to the ERAS/overnight protocol implementation on all levels and formed the treatment group (Overnight). This group involved a uniform goal towards an overnight stay with perioperative and ward nurses, allied health staff and hospital administration all aiming to achieve this outcome. In the other hospital, patients only had the anaesthetic and operative procedure as part of the Overnight protocol (surgeon/anaesthetist controlled elements). The remainder of the patient care team – nursing, allied health and hospital administration staff, did not specifically aim to achieve an overnight or short stay outcome for the patient's hospitalization. The perioperative measures detailed in the ERAS/Overnight protocol (Appendix S1) were not followed for the patients who formed the control group.

### Surgery

All TKRs were cemented and performed with a standard medial parapatellar approach, with the same posterior stabilized or medial pivot implants used in all patients. All THRs were performed using the SuperPATH approach with uncemented implants and a ceramic on polyethylene bearing.

### Overnight and control protocols

The overnight protocol and differences between the overnight and control groups are provided in Appendix S1, detailing preoperative, intraoperative and postoperative measures.

### Outcome measures

The primary outcome measure was LOS postoperatively, defined as the number of nights in the hospital after the surgery. The secondary outcome measures included early patient functional score improvements (preoperative to 6 week post‐operative Oxford Hip and Knee scores), patient satisfaction with their treatment (yes/no), adverse events (wound complications, falls during postoperative period, periprosthetic fracture, neurovascular injury, infection, dislocation, venous thromboembolism) and readmission into the hospital after discharge for any cause within 90 days of surgery.

### Statistical analysis

Normally distributed demographic data with continuous variables are reported as means and standard deviations with independent sample *t*‐tests. Categorical variables with skewed distributions such as LOS are reported as median and interquartile range (IQR) with median tests used to compare them. Frequency and percentages were reported for categorical variables and chi‐squared tests were done for their comparison. To make an accurate comparison of hospital LOS, we undertook a survival analysis approach where LOS in nights was used as the time variable and all subjects were considered to have an event at the end of LOS. Kaplan‐Meyer survival curves were generated to compare the LOS by hospitals for different sub‐groups. Statistical comparison of the curves was carried out by using log‐rank test.

### Ethics

Ethics Approval was obtained from Hunter New England Ethics Committee (Ref: 2020/ETH01441).

## Results

A total of 101 patients undergoing 56 THR and 55 TKR were included in the study across the two groups. The control group had 28 THR and 23 TKR patients. A summary of the demographic comparisons between the two groups are provided in Table 1.

**Table 1:** Comparison of pre‐operative demographics of the two groups

	Group				*P*‐value
	Overnight (*N* = 50)		Control (*N* = 51)		
Age‐Mean (*SD*)	60.6	(10.1)	63.7	(9.6)	0.127
BMI‐Mean (*SD*)	29.8	(4.8)	31.1	(4.7)	0.157
Sex					
Female *n*(%)	18	36.0%	27	52.9%	0.087
Male *n*(%)	32	64.0%	24	47.1%	
ASA					
1	0	0.0%	0	0.0%	0.001
2	40	80.0%	22	43.1%	
3	10	20.0%	28	54.9%	
4	0	0.0%	1	2.0%	
Diagnosis					
AVN	1	2.0%	1	2.0%	1.000
DDH	1	2.0%	1	2.0%	
OA	48	96.0%	49	96.1%	
Side of surgery					
L	26	52.0%	20	39.2%	0.226
R	24	48.0%	31	60.8%	
Type of surgery					
THR	28	56.0%	28	54.9%	
TKR	22	44.0%	23	45.1%	0.912

**Fig. 1:**
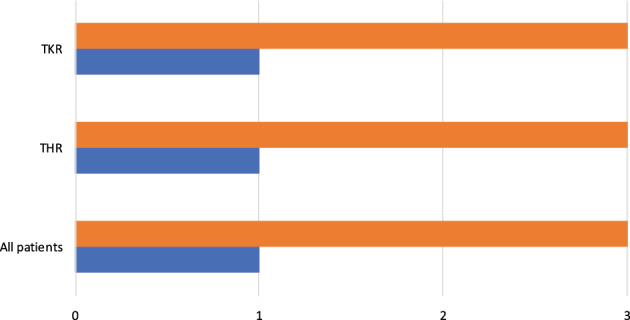
Comparison of hospital LOS. 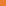 Control; 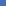 Overnight

Overall median LOS in the Overnight group was 1 day and significantly lower than the median LOS in the control group of 3 days (*P* < 0.0001) (Fig. 1). Sub‐analysis showed a median LOS of 1 day in the Overnight group and 3 days in the control group for THR (*P* < 0.0001). When comparing TKR, median LOS was also 1 day in the Overnight group and 3 days in the control group (*P* < 0.0001). Sub‐analysis comparing similar ASA grades showed similarly decreased median LOS in the Overnight group for ASA 2 patients (1 vs. 3 days, *P* < 0.0001) and ASA 3 patients (1 vs. 3 days, *P* < 0.0001).

**Fig. 2:**
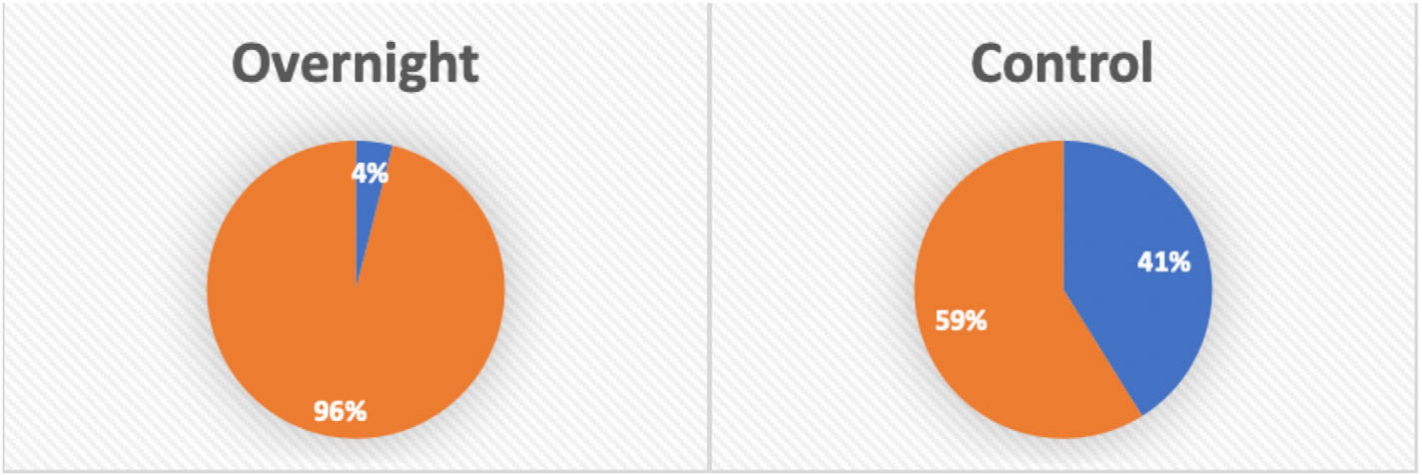
Comparison of patients utilizing inpatient rehabilitation. Overnight: 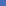 Inpatient Rehab; 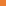 No Inpatient Rehab; Control: 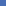 Inpatient Rehab; 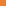 No Inpatient Rehab

41.2% of all joint replacement patients were discharged to rehabilitation facilities in the control group, compared with 4% in the Overnight group (*P* < 0.0001) (Fig. 2). Sub‐analysis showed 25% of control group patients and 0% of Overnight group patients who underwent THRs were discharged to rehabilitation facilities (*P* = 0.005). 60.9% of control group patients and 9.1% of Overnight group patients who underwent TKRs were discharged to rehabilitation facilities (*P* < 0.0001). Sub‐analysis comparing similar ASA grades showed decreased discharge to rehabilitation facilities in the Overnight group for ASA 2 patients (2.5% vs. 36.4%, *P* < 0.0001) and no significant difference in ASA three patients (10% vs. 42.9%, *P* = 0.06).

There were no significant differences in median VAS pain score between the two groups on D0 (Control‐VAS 2 vs. Overnight‐VAS 1, *P* = 0.195). There was a difference in median VAS pain score between the two groups on day of discharge from hospital (Control‐VAS 0 vs. Overnight‐VAS 2, *P* = 0.013). Sub‐analysis of THR and TKR showed significant differences in only the THR median VAS pain score on day of discharge from hospital (Control‐VAS 0 vs. Overnight‐VAS 2, *P* = 0.01).

Oxford Hip scores improved significantly from a mean of 8.68 preoperatively to a mean of 44.32 by 6 weeks postoperatively in the Overnight group, and from 13.79 to 45.54 in the control group. The mean improvement in Oxford Hip scores between the groups was 3.89 lower in the control group (*P* = 0.014). Oxford Knee scores improved significantly from a mean of 15.27 preoperatively to a mean of 42.05 6 weeks postoperatively in the Overnight group, and from 13.48 to 40.22 in the control group. The mean improvement in Oxford Knee scores between the groups was 0.03 lower in the control group (*P* = 0.987). All patients in both groups were satisfied with their treatment.

Four percent of patients had a blood transfusion in the control group compared with 0 in the Overnight group (*P* = 0.495). One patient in the Overnight group was readmitted with a complication‐requiring reoperation for wound dehiscence, after a fall 3 weeks postoperatively, compared with no patients in the control group (*P* = 0.495). There were no other complications or readmissions within 90 days in either group.

## Discussion

As recently as 2018, LOS following TKR and THR in Australia was still reported to average over 5 days,^11^, ^12^ although a recent single surgeon series comparing an ERAS protocol to general RACS/Medibank data did show improving median hip (two nights) and median knee (one night) LOS.^4^ Our study shows that an Overnight protocol with uptake by all stakeholders involved in patient care can safely and effectively allow overnight TKR and THR, with a median LOS of one night for both hips and knees in our Overnight intervention group.

Overnight or short‐stay joint replacement models have been an extremely topical issue in the last year or so. While being a standard of care in many centres in Europe and the United States,^13^, ^14^, ^15^, ^16^ it has largely been an uncommon practice in Australia due to a variety of factors. One such reason relates to the hospital remuneration based on days in hospital and health fund arrangements (case payments based on LOS and maximizing at a certain LOS, e.g. four nights). This then subconsciously or consciously flows on as a general attitude affecting all aspects of care^10^ including preoperative clinics setting patient expectations (for prolonged length of stay and need for inpatient rehabilitation), postoperative mobilization and group physiotherapy instead of individualized targeted physiotherapy, discharge planning aimed at day 4–5 discharge and inpatient rehabilitation bed availability also aimed to be available by this timeframe. Some private hospitals facilitate this attitude by introducing blanket physiotherapy classes, inpatient rehabilitation for all THR or TKR patients and creating a perception that staying longer will mean better access to rehabilitation and other services and in the patients' best interest. A culture and expectation amongst privately insured patients who have been paying for their policy, of ‘getting their money's worth by staying longer also perpetuates this attitude.

The need to involve all stakeholders to the Overnight protocol is critical in ensuring appropriate perioperative measures can be used to facilitate early patient recovery and discharge from hospital. This is evidenced in our study findings with a higher LOS in the control group of median 3 days, as well as a higher rate of inpatient rehabilitation in the control group of 41% (vs. 4% in Overnight group). The anaesthetic and operative Overnight measures were identical between the two groups, with the major difference to this higher median LOS being perioperative hospital uptake of Overnight measures. Similarly, the majority of patients who went to inpatient rehabilitation in the control group hospital had already been cleared for discharge home by physiotherapy, again reflecting hospital messages and subsequent patient expectations contributing to increased hospital LOS and use of inpatient rehabilitation facilities. The findings in this study on subgroup analysis of ASA 2 and ASA 3 patients also show that for the majority of patients, Overnight arthroplasty is feasible even with the presence of underlying comorbidities.

Cost savings associated with a successful Overnight model are substantial. The cost of a joint replacement as an Overnight patient without inpatient rehabilitation is estimated to be around AUD 13 000 (implant cost $9000, theatre cost $3000, inpatient hospital stay $1000) in this study, with this also including 6 weeks of rehab at home physiotherapy services. For the same procedure, the cost of a 5 day inpatient hospital stay ($21 000–$22 000)^17^ and a 2 week inpatient rehabilitation would cost over $30 000. The inpatient rehabilitation component of this alone would vary from $5000 to $17 000 based on variables such as different institutions and shared or single rooms. An estimated additional $4–6000 would be incurred for an outpatient day rehab programs of two sessions a week on an ongoing basis for up to 6 weeks. It is estimated that the economic fallout of recent lockdowns would be in the vicinity of $20 billion.^18^ The healthcare budget, like all other aspects of spending, is going to be under tremendous pressure and tight constraints will be palpable across both the private and public sectors. The cost differential referenced above would potentially increase the ability to provide these services within the allocated budgetary limits. Note: All costs are in Australian Dollars (AUD).

This study shows that overnight joint replacement surgery does not come at any increased risk to patient safety. This is also consistent with other studies on the subject.^19^, ^20^ The mildly increased pain VAS in the Overnight group (VAS 2 vs. 0 in control group) on day of discharge from hospital likely reflects the fact that these patients are going home on day 1 post‐surgery. Nevertheless, this slight increased pain score did not affect patient satisfaction or short‐term functional recovery. Our study shows that overnight joint replacement surgery following an Overnight protocol leads to similar functional improvements following THR and TKR as evidenced by marginally higher (but below the minimal clinically important change) functional score improvements in the Overnight group.

These findings on the effectiveness of a multi‐disciplinary Overnight protocol in safely decreasing hospital LOS following THR and TKR to overnight, should be interpreted while considering the study limitations. The different patient populations undergoing surgery at the two hospitals had different ASA grade distributions with more ASA 2 patients in the Overnight group and more ASA 3 patients in the control group. We acknowledge that the Overnight protocol and overnight stay joint replacement surgery is not for all patients. Certain individual patients may require additional support following their surgery, more nights in hospital and access to inpatient rehabilitation. This should be offered on an as needs basis considering individual patient factors, rather than the blanket inpatient rehabilitation pathways for all patients. However, the subgroup analysis showed significant differences between the two hospitals even when comparing only ASA 2 patients, and a trend towards similar findings in ASA 3 patients although this did not reach statistical significance. Furthermore, the majority of patients from the control group who went to inpatient rehabilitation had already been cleared for discharge home by physiotherapy prior to their transfer to the rehabilitation service. This may reflect potential payment incentives guiding patient care. Secondly, a formal cost analysis was not within the scope of this study. However, we feel the extrapolation of hospital LOS reduction and decreased use of inpatient rehabilitation facilities presents sufficient evidence on the cost saving benefit of our multi‐disciplinary Overnight protocol.

Overnight THR and TKR can safely be performed in the majority of patients, with a multi‐disciplinary approach Overnight protocol and involvement of all perioperative stakeholders.

## Conflict of interest

None declared.

## Author contributions

**Jason Chinnappa:** Conceptualization; formal analysis; investigation; methodology; supervision; validation; writing – original draft; writing – review and editing. **Sam Aktas:** Data curation; project administration; writing – original draft. **Abdul Majid Dabboussi Majid Dabboussi:** Data curation; investigation; project administration; writing – original draft. **Md Bayzidur Rahman:** Data curation; formal analysis; methodology; writing – review and editing. **Sol Qurashi:** Conceptualization; methodology; project administration; resources; writing – original draft; writing – review and editing.

## Supporting information


**Appendix S1** ERAS Protocol.
