# Supplementary material for: Overnight joint replacement surgery: a pilot Australian study
Source: ANZ J Surg. 2022 Aug 15;92(10):2683–7. doi: 10.1111/ans.17977 (PMC9804704; doi:10.1111/ans.17977)
Supplement: Supplementary file 1 — Appendix S1 ERAS Protocol. [file ANS-92-2683-s001.docx]

**Appendix A: Overnight Stay Protocol**

**Preoperative**:

(Control group only)

- Preoperative Joint Replacement Clinic (generically run by hospital nursing and allied / discharge planning staff for all joint replacements patients under all appointed surgeons)

(All patients)

- Preoperative Joint Replacement education/counselling in the clinics by the Chief operating surgeon to the patient and their immediate family / partner.
- Preoperative assessment done by the anaesthetist in charge to discuss the anaesthetic walk through for the procedure.
- Premedication-Oral analgesic was given preoperatively. Avoid sedatives (Benzodiazepines, Neuroleptics or Opioids given preoperatively)
- Preoperative fasting of 6 hours with clear fluids allowed till 2 hours preoperatively. Preoperative carbohydrate loading.
- Preoperative warming of patient with warmers and blankets was implemented from ward to preoperative anaesthetic bay.

**Intraoperative**: (All patients)

- General anaesthesia as a standard approach. No nerve blocks that impair motor function and early mobilisation.
- Minimum use of opioids.
- Drugs used:
  - Midazolam
  - Propofol for induction then TCI
  - Fentanyl approx. 300mcg for entire case (50mcg intubation, 100mcg pre incision, 100mcg during, 50mcg prior to wake up)
  - Vecuronium 20mg intubation and 10mg bolus as required during case
  - Paracetamol 1g then chart QID for 5 days post operative
  - Parecoxib 40mg then chart NSAID for 3-5 days post operative
  - Tramadol 200mg pre incision
  - Ketamine 50mg pre incision
  - Dexamethasone 8mg
  - Ondansetron 8mg and chart PRN post operative
  - If other indicators for PONV then add metoclopramide 20mg and cyclizine 50mg
    Sugammadex 200mg
- 1g Tranexamic Acid IV 15 minutes before incision followed by 2 doses post-operatively at 8 and 16 hours post-operation
- Cefazolin 2g post intubation then chart 3 x doses q8h post operative
- IVF 1L intraop with second litre started before end of case. 3 x 1L post op (q5h, q8h, q12h)
- Continued intraoperative warming by warmed IV infusion & air warming.
- No indwelling catheters and no drains during or after surgery.
- Application of intermittent pneumatic compression device (IPCD) to the leg opposite to surgical side.
- Local Infiltrative Analgesia (LIA) of Ropivacaine (0.2%) + adrenaline after arthrotomy, during the procedure and closure.

**Postoperative**: (All patients)

- No PCA/ No IDC (tethers patient to bed and increases risk of infection)
- Discontinue IV fluids after surgery when the vital parameters are stable. Start oral feeds early.
- Antiemetic prophylaxis.
- Ice packs for 30 minutes every 2 hours as cryo-compression.
- DVT Prophylaxis with intermittent pneumatic compression devices (IPCD), compression socks and enoxaparin (LMWH) SC injections for 10 days post-operation.
- Multimodal analgesia: Regular Paracetamol + NSAID, Tapentadol SR 50mg BD for 3-4 days and Tapentadol IR 50mg q4h PRN. Oxycodone for PACU 1mg q5mins max 5-8mg depending on patient
- Patient goes home with post op pain sheet and scripts
- Early postoperative (recovery room) oral carbohydrate supplementation
- Patients are reviewed on ward end of list to ensure they have mobilized

Mobilisation goals: (Overnight Group)

- Physiotherapy initiated on day of surgery. Patients were made to walk with a walking frame then progress to crutches, if safe, on the day of surgery. Mobilisation Protocol: Mobilisation within 6 hours emphasized as well as one on one sessions. More than one physiotherapist directed mobilisation on Day 0. Physiotherapist stayed till late or shifts staggered to ensure evening mobilization and
- POD 0: Assisted walking, bed to chair transfer, Independent walking (with gutter crutches), independent transfers.
- POD 1: Independent walking (with gutter crutches), stair climbing .

Mobilisation goals: (Control Group)

- Physiotherapy initiated on day of surgery. Patients were made to walk with a walking frame on the day of surgery.
- Mobilisation Protocol: Mobilisation within 6 hours emphasized but often patients would not be mobilsed till later, and often by surgeon or nursing staff as no physiotherapy cover. Only one physiotherapist directed mobilisation on Day 0. From Day 1 onwards, more group based focus instead of one on one sessions.
- Discharge Criterion (Overnight Stay Group)
- Early hospital discharge (Aimed at Day 1). Discharge criteria was identified to be when the patient mobilized independently, was able to climb stairs and do an independent bed to chair transfer, provided medical indices were normal and patient comfortable. Ultimate discharge decision by Surgeon.
- Discharge Criterion (Control Group)
- No fixed Hospital Discharge date objective. Directed by discharge planning staff. Not dependent on clearance by physiotherapist or medically fitness (could stay even if cleared medically).
- Patients encouraged to participate in group physiotherapy sessions.
- Patients encouraged to consider inpatient rehabilitation if covered by health fund.
- Ultimate discharge decision by discharge planning staff.
